# Comparative expression pathway analysis of human and canine mammary tumors

**DOI:** 10.1186/1471-2164-10-135

**Published:** 2009-03-27

**Authors:** Paolo Uva, Luigi Aurisicchio, James Watters, Andrey Loboda, Amit Kulkarni, John Castle, Fabio Palombo, Valentina Viti, Giuseppe Mesiti, Valentina Zappulli, Laura Marconato, Francesca Abramo, Gennaro Ciliberto, Armin Lahm, Nicola La Monica, Emanuele de Rinaldis

**Affiliations:** 1 IRBM P. Angeletti, Merck MRL-Rome, Via Pontina Km, 30600 Pomezia, Italy; 2 Department of Molecular Profiling, Merck Research Laboratories, West Point, Pennsylvania, USA; 3 Rosetta Inpharmatics LLC, a wholly owned subsidiary of Merck and Co Inc., Terry Avenue North, Seattle, WA 98109, USA; 4 Department of Public Health, Veterinary Hygiene and Comparative Pathology, University of Padua, Italy; 5 Clinica Veterinaria L'Arca, Vico Cacciottoli 46/47, 80129 Naples, Italy; 6 Department of Animal Pathology, University of Pisa, Italy

## Abstract

**Background:**

Spontaneous tumors in dog have been demonstrated to share many features with their human counterparts, including relevant molecular targets, histological appearance, genetics, biological behavior and response to conventional treatments. Mammary tumors in dog therefore provide an attractive alternative to more classical mouse models, such as transgenics or xenografts, where the tumour is artificially induced. To assess the extent to which dog tumors represent clinically significant human phenotypes, we performed the first genome-wide comparative analysis of transcriptional changes occurring in mammary tumors of the two species, with particular focus on the molecular pathways involved.

**Results:**

We analyzed human and dog gene expression data derived from both tumor and normal mammary samples. By analyzing the expression levels of about ten thousand dog/human orthologous genes we observed a significant overlap of genes deregulated in the mammary tumor samples, as compared to their normal counterparts. Pathway analysis of gene expression data revealed a great degree of similarity in the perturbation of many cancer-related pathways, including the 'PI3K/AKT', 'KRAS', 'PTEN', 'WNT-beta catenin' and 'MAPK cascade'. Moreover, we show that the transcriptional relationships between different gene signatures observed in human breast cancer are largely maintained in the canine model, suggesting a close interspecies similarity in the network of cancer signalling circuitries.

**Conclusion:**

Our data confirm and further strengthen the value of the canine mammary cancer model and open up new perspectives for the evaluation of novel cancer therapeutics and the development of prognostic and diagnostic biomarkers to be used in clinical studies.

## Background

The availability of predictive preclinical animal models for human breast tumours represents a major challenge in breast cancer research. *In viv*o mouse models such as xenografts and transgenics, although widely used, have been demonstrated to fail in recapitulating essential features of human breast cancers such as heterogeneity, tumour microenvironment and dependence on steroid hormones [1,2]. Besides the intrinsic evolutionary distance between mice and humans, additional differences can originate from induced genetic modifications (transgenic mice) or from the altered presence of adjacent normal tissue, stromal cells, vasculature and immune system components (xenografts) [3-7]. Together these factors translate into a limited value of these mouse models for the study of cancer pathogenesis, progression and therapy, and represent a major obstacle to the identification of reliable predictive molecular biomarkers and the development of effective therapeutic agents [1,8].

However, during the last few years, along with the sequencing of the entire dog genome (99% complete, ~2.5 billion base pairs) and the clear evidence of its close similarity with the human genome [9], the dog has emerged as an attractive alternative model for cancer research [9]. For many gene families and in particular those associated with cancer, the similarities between dog and human gene sequences have been found to be much closer than the respective counterparts in mouse [10]. Molecular cytogenetic analysis of canine tumour cells derived from haematological malignancies revealed the retention of ancestral chromosomal aberrations in comparable cancers of human and dog [11,12]. In mammary carcinomas, altered expression of the *ERBB2 *and *TP53 *genes were observed to be similar in the two species, suggesting similar roles in carcinogenesis and potential use as prognostic indicators [13-15]. It was also observed that similar mutations in oncogenes can result in different cancers in humans and dogs. For example, similar mutations in *KIT*, a tyrosine kinase growth factor receptor, have been identified in both human gastrointestinal stromal tumours (GIST) and dog mast-cell cancers [16]. Moreover, comparative histological analyses indicate that the intratumoural (cell-to-cell) heterogeneity observed in human breast tumours also occurs in the cognate dog tumors [17]. The natural consequences of this heterogeneity underlie the deadly features of human cancers, namely acquired resistance to therapy, recurrence and metastasis.

Additional and more general considerations have contributed to the increased interest in the dog as a preclinical model. Dogs have a large body size and are relatively outbred as compared to other laboratory animals, therefore providing a genetic diversity similar to that seen in humans [9]. Spontaneous cancers in dogs develop in the context of a natural immune system where the tumor and its microenvironment are syngeneic. Unlike mice, dogs share a common environment with humans and are exposed to some of the same carcinogens [18]. Moreover, compared to humans, the shorter life span of dogs facilitates the study of mammary tumours that develop after a few years instead of decades. Finally, as in humans, progesterone treatment, advancing age, obesity and diet, represent risk factors for mammary tumour development in dogs [9].

In this study we present the first genome-wide analysis of transcriptional changes occurring in the mammary tumours of dog, including a comparative analysis with respect to human breast tumors. Starting from independent human and dog microarray studies, changes in gene expression levels were compared following the mapping of the orthologous genes represented across both array platforms. By unsupervised analysis, we show that clustering is predominantly driven by the origin of the samples (tumour/normal) rather than by species (dog/human), indicating a close overall transcriptional similarity between tumours in both systems. A significant number of genes differentially expressed in human breast tumours, compared to normal human samples, were also found deregulated in the canine model. Moreover, a pathway-focused analysis of these genes revealed a large degree of similarity in the up- and down-regulation of several cancer related pathways.

We have also addressed the comparison of human and dog tumours from a more systemic perspective, by exploring the networks of transcriptional relationships existing between different gene signatures. Various prognostic and oncogenic signatures, derived from independent human breast cancer studies, were collected from the literature and their expression relationships were examined in human breast tumours. We show that many of these signatures, although developed within different experimental settings and contexts, exhibit coordinated patterns of expression. By performing the same analysis on the dog tumour samples, we observed that these relationships are largely maintained, thus suggesting a close interspecies similarity in the network of cancer signalling circuitries governing the establishment and the progression of the tumour.

Collectively our data confirm and strengthen the value of the dog as a suitable model for studying breast cancer, including the development of prognostic molecular biomarkers and the evaluation of novel cancer therapeutics.

## Results

### Data

The dog gene expression data set consists of microarray data from 33 mammary samples, comprising 7 healthy and 26 diseased samples. All samples were extracted from a total of 10 animals, each animal bearing one or more tumors.

All dog carcinomas were assigned to grade 1, according to diagnostic criteria proposed by the World Health Organization (WHO) [19]. For the purpose of the study, canine samples were grouped in four classes (from 0 to 3), representing increasing levels of aggressiveness (see Additional file 1), as follows. ***Class 0***: 7 normal samples; ***class 1***: 3 'hyperplastic/dysplastic lesions' (representing benign proliferative lesions) and 2 'benign tumors'; ***class 2***: 2 '*in situ *carcinomas' (malignant tumor with a better prognosis as compared to other malignant lesions) and 2 'tubular carcinomas' (showing well differentiated morphology and no evidence of infiltration); ***class 3***: 6 'simple carcinomas' and 11 'complex carcinomas' (representing aggressive forms of malignant tumors). For simplicity, classes 0, 1, 2 and 3 are respectively, referred to in the text as 'normal', 'benign', 'intermediate' and 'malignant' samples.

The human data set consists of 129 samples, including 68 malignant tumors and 61 matched adjacent normal samples. Human tumors were classified according to TNM classification [20] in stages 1–4, with 3 tumor samples belonging to stage 1, 51 to stage 2, 13 to stage 3 and 1 to stage 4 (see Additional file 2).

Microarray experiments were run on two different custom designed, double channel Agilent microarray platforms (25K probes for human and 44K probes for dog). Both data sets used a pool of normal samples as a common baseline sample, respectively derived from human and dog samples (see Methods). For each sample, gene expression ratio values were calculated by comparing the individual sample (tumor or normal) with the respective common baseline.

### Gene expression analysis of dog mammary tumours

**Figure 1:**
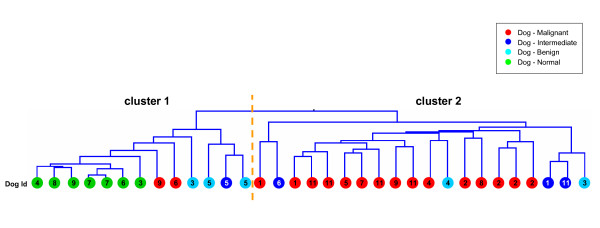
**Unsupervised hierarchical clustering of dog samples**. Unsupervised hierarchical clustering of 33 dog samples based on a subset of 2008 most variable genes selected using a filter of three-fold change or more on at least three samples. Samples are colored according to pathological classification and numbered according to the individual dog origin.

**Figure 2:**
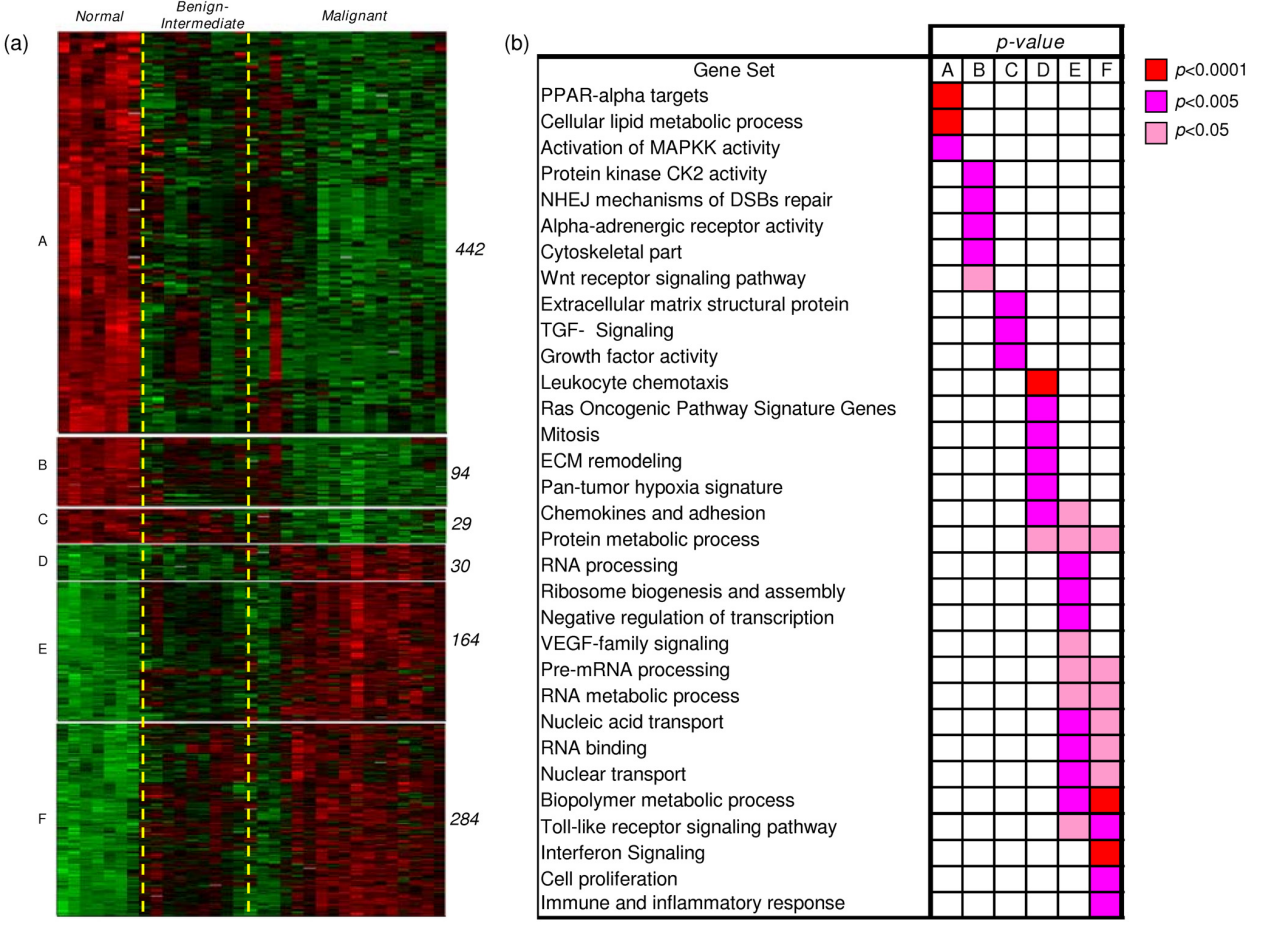
**Identification of gene classes in canine mammary samples**. **(a) **Hierarchical clustering of dog samples based on a subset of 1043 genes selected by 1-way ANOVA (q-value < 0.001). For display purposes, samples in each class (normal, intermediate-benign, malignant) were clustered separately and arranged from normal (left) to malignant (right). Genes were grouped in six groups according to their pattern of expression (see description in the text) and hierarchically clustered separately. The gene size of each group is indicated to the right of each cluster. **(b) **Graphical representation of the significance of gene set enrichment for the six gene groups described in (a). Enrichment scores are computed by one-sided Fisher's exact test, using the entire set of orthologous genes represented on both human and dog microarray platforms as the reference population.

To characterize the overall diversity between individual dog samples, we performed an unsupervised hierarchical clustering of all 33 dog samples, arbitrarily assigned to three major classes: 'normal', 'benign and intermediate tumors' and 'malignant tumors' (Figure 1). The first bifurcation of the hierarchical clustering dendrogram identifies two clusters of samples, which represent non-random distributions of normal and malignant samples from the complete population. Normal samples are over-represented in cluster 1 (hypergeometric test; *P *= 4 × 10^-5^), segregating into the same sub-cluster, while malignant tumors are over-represented in cluster 2 (hypergeometric test; *P *= 1.1 × 10^-3^). Interestingly, samples from the 'benign and intermediate tumor' class were evenly distributed across the two clusters, as expected by their intermediate phenotype falling between 'normal' samples and 'malignant tumors'. Genes differentially expressed in the three sample groups were then identified by ANOVA analysis. Using a conservative q-value threshold of 0.001, a list of 1043 genes was selected (see Additional file 3). According to their patterns of expression, six groups of genes could be identified (Figure 2). Each group was analyzed separately by gene set enrichment analysis (see Methods and Additional file 3).

#### Group A: 442 genes down-regulated in 'benign-intermediate tumors' and 'malignant tumors' as compared to 'normal'

Genes in this group are enriched for lipid metabolism related genes (P = 1e-5), PPAR-alpha targets (P = 3e-6) and genes related to activation of the *MAPK *pathway (P = 0.0028), including the *ELK3 *transcription factor. According to our data (see below) and previously reported data [21], ELK3 expression is also decreased in human breast carcinoma. Moreover, ELK3 down-regulation was observed in malignant mesothelioma [22] and cervical cancer cells [23], thus suggesting a potential role as a tumor suppressor.

#### Group B: 94 genes down-regulated in 'malignant tumors' as compared to 'normal'

This group is enriched for genes involved in protein kinase CK2 activity (*P *= 0.0002), double stranded break repair (*P *= 0.0004), constituents of cytoskeleton (*P *= 0.0002) and WNT receptor signaling pathway (*P *= 0.0070). Amongst them are two genes of the CK2 complex: the CK2beta regulatory subunit, reported to inhibit cell proliferation [24] and the CK2alpha2 catalytic subunit (*CSNK2A2*) [25].

#### Group C: 29 genes up-regulated in 'normal' and 'benign-intermediate tumors' as compared to 'malignant tumors'

This group is enriched for structural components of the extra-cellular matrix (ECM) (*P *= 0.0003) and growth factor activity (p = 0.0048) related genes represent the dominant feature of this group. In humans, the expression of ECM genes in breast cancer is known to be related to the clinical outcome [26]. The lower expression of structural components of the ECM in tumors, coupled with the increased expression of genes involved in ECM remodeling observed in group D (see below), is likely to contribute to the creation of a favorable microenvironment for tumor invasion. Growth factor related genes down-regulated in tumour samples are: *BMP4*, found to inhibit the tumorigenic potential of human brain tumor initiating cells[27]; *MAP2K2*, a regulator of G1/S transition [28] and *LTBP4*, reported to be down-regulated in human mammary adenocarcinoma [29];

#### Group D: 30 genes up-regulated in 'malignant tumor' as compared to 'benign-intermediate tumors' and 'normal'

A significant overlap was observed with genes of the KRAS oncogenic pathway, (P = 0.0036), the hypoxia signature (P = 0.0012), ECM remodeling (P = 0.0002) and chemokines and adhesion (P = 0.0003). Genes in the KRAS oncogenic pathway include interleukin-8 (*IL*8), a member of the CXC chemokine family and a key player in the activation of the inflammatory response. *IL8 *is secreted by a large number of tumors, including breast [30], and its expression is reported to be modulated by hypoxia [31-33].

#### Group E: 164 genes up-regulated in 'malignant tumor' as compared to 'normal'

This group is enriched for genes associated with increased proliferative activity such as RNA processing (P = 0.0014), RNA binding (P = 0.0013), ribosome biogenesis (P = 0.0016). VEGF-family (P = 0.0069) and Toll-like receptor (P = 0.0445) signaling pathways were also found to be enriched in this set. The VEGF-family signaling pathway is activated in tumors, well known to induce the formation of new blood vessels and its inhibition leads to the stasis or even regression of the tumor [34]. The Toll-like receptor (TLR) signaling pathway is known to promote the malignant transformation of normal epithelial cells in various tumors (reviewed in [35]).

#### Group F: genes up-regulated in 'malignant tumors' and 'benign-intermediate tumors' as compared to 'normal' (284)

This group shares many of the enrichments observed in the previous group E, indicating that the major biological "themes" distinguishing malignant from normal tissues are also present in the less aggressive tumors represented in the "benign and intermediate" class. In addition to group E, this group was found enriched in cell proliferation genes (P = 0.0046), genes responsible for the immune and inflammatory response (P = 0.0020) and genes involved in interferon signaling (P = 7e-5). The list includes eight genes known to be modulated by interferon levels in human: *CXCL16, IFI44, IFNAR1, IFNAR2, IFNGR2, ISG15, MX1*, and *STAT2*. Specific enrichment of immune-related genes in group F probably reflects the presence of lymphocytes infiltrating both "benign and intermediate" and "malignant" tumor classes as compared to their normal tissue counterparts. However, as previously reported, a significant proportion of these genes might also originate in breast tumor cells and not be due exclusively to stromal infiltration [36].

### Unsupervised clustering analysis of dog and human samples

The overall similarity between dog and human samples was analyzed using the unsupervised principal component analysis (PCA) of dog and human samples. The analysis was performed including the expression values of 9.963 genes identified as orthologous between the two species and represented on the respective microarray platforms (see Methods).

**Figure 3:**
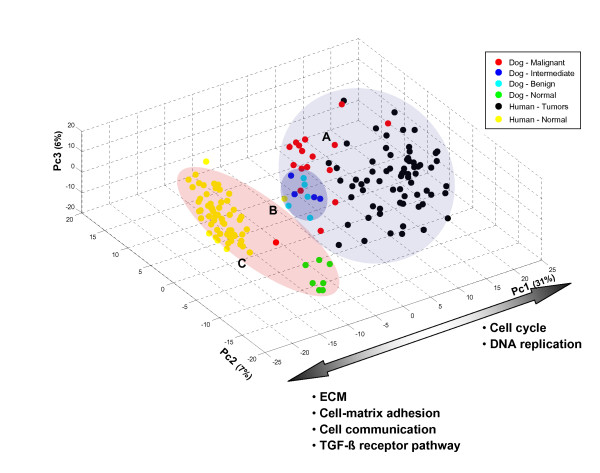
**Principal component analysis of human-dog combined dataset**. Results of principal component analysis based on the subset of 9,963 human-dog orthologous genes. The 162 samples were projected on the first three principal components that capture ~44% of the total variability. Three main sample clusters are highlighted in colors. Cluster A contains the majority of human and dog tumors. Cluster B is a sub-cluster of cluster A and contains mostly 'benign' and 'intermediate' dog tumors. Cluster C includes almost all normal samples from human and dog. The genes contributing the most to the first principal component analysis were analyzed by genes set enrichment analysis and enriched gene sets (p < 0.05) were selected. Results are summarized along the grey arrow. Among the others, cell cycle (P = 6e-9) and DNA replication (P = 7e-6) emerged as enriched gene sets (up-regulated in both species tumors and listed on the right side of the arrow), as well as extracellular matrix (P = 0.0025), cell matrix adhesion (P = 0.0078), cell communication (P = 0.0207), TGF-β receptor signaling pathway (P = 0.0115) (down-regulated in both species tumors and listed on the left side of the arrow).

All 162 samples from the two species were projected onto the first three principal components (Figure 3). The first component captures most of the variability (31%) and drives the separation of tumor and normal samples of both species. An additional 7% and 6% of the total variability is captured by the second and the third principal components, respectively, which instead drive the separation of groups of normal and tumor samples within each species. As evident from Figure 3, general differences in tumor/normal characteristics of the samples dominates with respect to the dog/human species membership. The species separation is however clearly evident within the group of normal samples while being less pronounced in the group of tumors, most likely because of the higher heterogeneity of these samples as compared to healthy normal tissue. Noticeably, one canine (M6b) tumor clusters in the group of normal samples. In absence of additional information, this could supposedly be explained by a particular cellular composition of this sample, where the epithelial compartment is under-represented with respect to the stromal part.

To identify the most relevant biological 'themes' driving the separation of tumor and normal samples and represented on the first PC, genes most correlated with this axis were analyzed by gene set enrichment analysis (Additional file 4 and Figure 3).

Consistent with the previous analysis run on dog samples only, genes with higher expression in tumors (positive correlation with first PC) were found to be enriched for cell cycle (P = 6e-9) and DNA replication (P = 7e-6) related genes.

Conversely, genes with higher expression in normal samples (negative correlation with first PC) were enriched for genes related to the extracellular matrix (P = 0.0025), cell matrix adhesion (P = 0.0078), cell communication (P = 0.0207) and TGF-β receptor signaling pathway (P = 0.0115). Interestingly, the nine dog tumors classified as 'benign' and 'intermediate' cluster somewhere in between tumor and the normal samples in the PCA space, along the first principal component. This observation is consistent with the biology of low aggressive tumors, which maintain some of the biological features of the normal tissue counterpart such as low proliferation rate and limited vascularization.

### Comparative analysis of deregulated genes and pathways in human and dog mammary cancer

**Figure 4:**
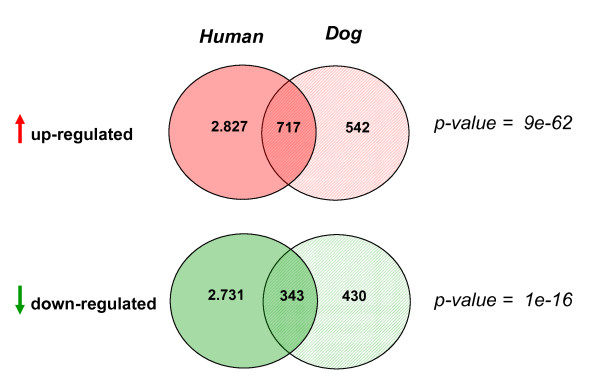
**Up and Down regulated genes in Human and Dog**. Venn diagrams representing up- and down-regulated genes in human and dog mammary tumors. On the right side, the Fisher's exact test p-values of the overlaps are reported.

**Figure 5:**
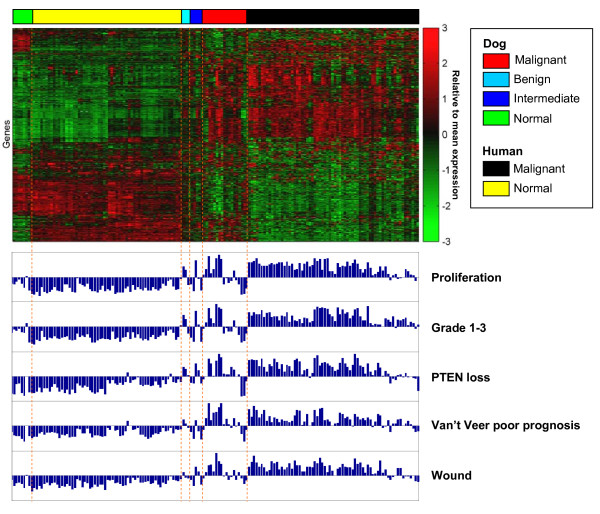
**Hierarchical clustering of human-dog combined dataset**. Clustering of the combined gene expression data for 33 dog (17 malignant, 5 benign and 4 intermediate) and 129 human (68 malignant and 61 normal) breast samples. A subset of 600 most variable genes was selected (see Methods for details). Samples in each class (dog normal, benign, intermediate and malignant samples, and human normal and malignant samples) were hierarchically clustered separately based on the Pearson correlation coefficients, and then columns were ordered based on class membership. Genes were hierarchically clustered based on Pearson correlation coefficients. The histograms at the bottom represent for each sample (column) the respective signature score values [37-41] (see Methods).

By using the Student's t-test we separately identified for human and dog, those genes globally deregulated in tumors as compared to their normal counterparts (Figure 4 and Additional file 5). To allow for a consistent comparison between human and dog, samples classified as 'benign' or 'intermediate' were excluded from the t-test analysis of the dog data set. Out of the set of 9.963 orthologous genes, 1.259 genes were identified as significantly up-regulated in dog tumors (q-value < 0.001) and many of them (717, hypergeometric test p = 9e-62) were also up-regulated in human tumor samples. Similarly, of the 773 genes down-regulated in dog tumors, a significant number (343, hypergeometric p = 1e-16) were also down-regulated in the human tumour samples. Thus, the analysis reveals the presence of a shared core of genes commonly deregulated in both human and dog mammary tumors. Although statistically significant, we believe this number of 'core genes' represents only a conservative estimate. This is most likely due to the limited size of the dog data set, which results in the reduced power of the t-test used for the identification of differentially expressed genes. The large overlap between genes deregulated (either up or down) in dog and human can also be appreciated in a heat map representation including data from both species, after hierarchical clustering (Figure 5) [37-41].

By gene set enrichment analysis we attempted to identify those gene sets or pathways showing a global deregulation in tumor samples as compared to their normal counterparts (see Methods).

**Figure 6:**
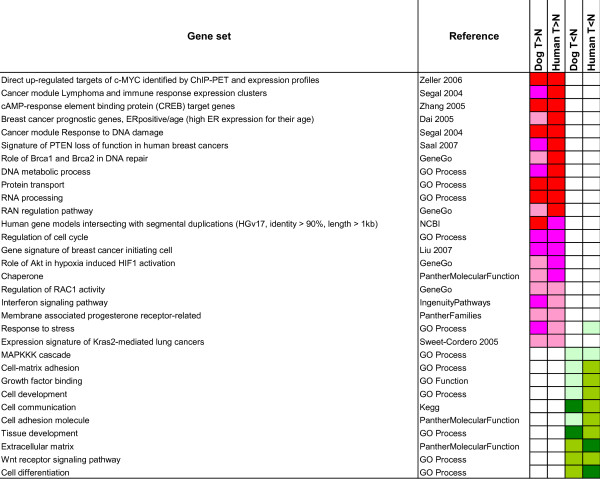
**Gene set enrichment analysis of tumor deregulated genes**. Results of gene set enrichment analysis carried out on the tumor deregulated genes independently identified in dog and human datasets. Enrichment p-values are computed using the Fisher's exact test. A selection of the gene sets enriched (p < 0.05) in both human and canine datasets is shown.

We defined as "up-" or "down-regulated" those gene sets or pathways statistically enriched in the list of genes up- or down-regulated, respectively, in tumors (the complete results are available in Additional file 6). As expected, a large part of the pathways showing up-regulation in tumors from both species are related to increased proliferating activity and to the general reorganization of cells observed in many tumor types (Figure 6). In particular, we observed gene sets related to cell cycle (S phase, M/G1, G1/S), protein and RNA transport (including RAN mediated), nucleotide and nucleic acid metabolism and transport, DNA repair and metabolism.

Conversely, gene sets and pathways related to cell and tissue development, cell matrix adhesion and cell communication emerged as being down-regulated. These findings are consistent with the notion that silencing of these processes favors tumour cell invasion and migration by coordinately impairing cell differentiation, adhesion to the extracellular matrix and cell-cell communication [42,43]. More specific sets of genes emerging as perturbed in both human and canine mammary tumors are described in more detail below.

### Commonly up-regulated gene sets/pathways

#### Gene signature of breast cancer initiating cells

a group of genes identified as being differentially expressed in a population of tumor initiating cells (CD44+CD24-) as compared to normal breast epithelium [44]. The up-regulation of this gene set provides indirect evidence of the presence of tumor initiating cells in both species tumor samples. If confirmed by experimental validation, this result would promote dog as a suitable model also for the study of breast tumor initiating cells.

#### Expression signature of KRAS2 mediated lung cancer

KRAS2 (*KRAS*) is a signal transduction GTPase, turned permanently on by somatic mutation in many cancers, including breast [45,46]. This group of genes was found to be controlled at the transcriptional level by signaling events downstream of the activated form of the *KRAS *oncogene [45]. The activation of this gene set is consistent with the observed up-regulated levels of *KRAS *in tumors from both species and with the up-regulation of pathways such as 'PI3K/AKT' downstream of *KRAS *(see below).

#### Signature of PTEN loss of function in human breast cancers

the 'PTEN loss of function' signature was identified in human breast tumors lacking *PTEN *protein expression [47]. Since loss of *PTEN *implicates strong activation of the 'PI3K/AKT' pathway, the up-regulation of this group of genes as a whole represents a marker of the activation of the 'PI3K/AKT' pathway. This pathway is an important regulator of cell proliferation and survival and its aberrant activation has been associated to the development and progression of a number of cancers [47].

#### Role of AKT in hypoxia induced HIF1 activation (PI3K/AKT)

amongst many cellular processes, the 'PI3K/AKT' pathway [48] activates a number of genes involved in the activation of HIF1, with direct *HIF1A *activation preceding the hypoxia-induced activation that only occurs when the growing cells outstrip the available oxygen supply. *HIF1A*, up-regulated in both tumor types, is a central player in the adaptation to hypoxia and known to be frequently activated in different tumors [48].

#### Cancer module Lymphoma and immune response expression clusters and Interferon signaling

although the up-regulation of these gene sets could be the consequence of lymphocyte infiltrates present in the samples, the intrinsic up-regulation of immune related genes in the tumor itself has also been reported [36].

#### Regulation of RAC1 activity and RAN regulation pathway

*RAN *and *RAC1 *are two other members of the GTPase KRAS superfamily and, like *KRAS*, are involved in many cellular processes, including the cell cycle, cell-cell adhesion, motility and of epithelial differentiation [49]. The observed up-regulation of this gene set, together with the activation of the *KRAS *activity signature, reiterates the important roles in tumor development and maintenance for different members of the KRAS superfamily of both species.

### Commonly down-regulated gene sets/pathways

#### WNT-receptor signaling pathway (WNT-beta catenin)

this pathway describes a complex network of proteins known for their important role in embryogenesis and normal physiological processes in adult animals and with a central role in cancer [50]. Although the pathway is known to be globally activated in many tumors [50] here we observe – consistently both in human and dog – a down-regulation of many pathway components. However, a closer look at the respective genes reveals that many of them (*SFRP5 *[51], *WISP3 *[52], *DKK4 *[53], *FRZB *[54] and *JUP *[55]) are antagonists of the 'WNT-beta catenin' pathway, thus exerting a tumor suppressor role in normal conditions. Down-regulation of the antagonistic components of the 'WNT-beta catenin' pathway in breast cancer has been reported [50,56]. Interestingly, *WNT4 *and *WNT5A *are down- and up-regulated in both species, respectively. This inverse deregulation has been observed to be associated with the epithelial-mesenchymal transition in human squamous carcinoma cells, a developmental event central to cancer progression [57].

#### MAPKKK cascade

mitogen-activated protein (*MAP*) kinases are serine/threonine-specific protein kinases that respond to extracellular stimuli and regulate various cellular activities, such as gene expression, mitosis, differentiation, and cell survival/apoptosis [58]. The *MAPK *signaling pathway involves a complex network of interactions between proteins, including the activation of the KRAS protein and the inactivation of various components of the cellular apoptotic machinery. The following genes were observed down-regulated in both dog and human tumors: *ELK3*, known to play a role in the activation of *MAPK *activity and decreased mRNA expression was already observed in breast cancer cells [21,59,60]; *CNKSR1 *known to act as tumor suppressor in *KRAS*-induced apoptosis and to negatively regulate cell proliferation [61]; *GPS2*, a G protein pathway suppressor which modulates cellular signaling pathways and enhances p53-induced apoptosis [62]; QARS, a component of aminoacyl-tRNA synthetase multienzyme complex which exerts an anti-apoptotic effect by binding and inhibiting *MAP3K5*, a kinase that plays a key role in apoptosis [63].

### Analysis of relationships between the expression patterns of prognostic and oncogenic signatures

**Figure 7:**
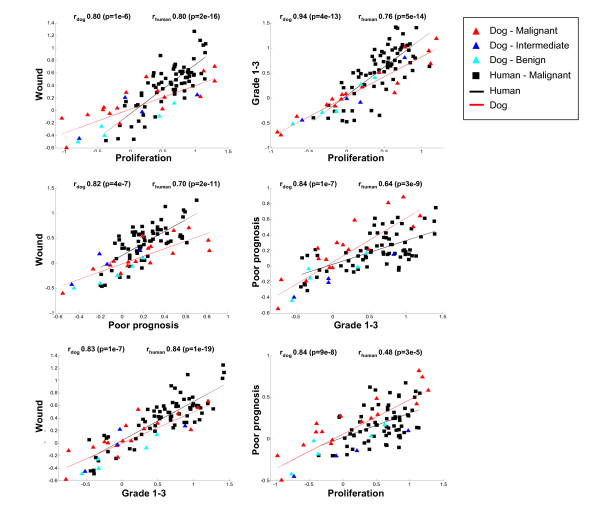
**Relationships between prognostic signatures**. Scatter plots showing the pair wise relationships between the prognostic signatures. For each signature the score was calculated as the weighted average of the expression level of the genes in the signature (see Methods). To compensate for the possibility that different signatures might show high correlation merely as a result of a common gene composition, the overlapping genes between each signature pair were not taken into account in the computation of the scores. Each point in the scatter plots represents a single tumor analyzed in the human and the dog dataset, respectively represented by squares and triangles. The Pearson's correlations and the corresponding p-values between each pair of expression signatures across the human (n = 68) and canine (n = 26) tumor datasets are indicated in each panel.

To get a comprehensive view of possible relationships existing between various cancer related signatures in the human and dog mammary tumors, we closely analyzed a large and heterogeneous compendium of published transcriptional signatures, all related to breast cancer. The signatures composing the compendium were defined in independent studies using different methodologies and cancer models, including human primary breast tumors, tumors from transgenic mice, and *in vitro *cell lines (Table 1) [37-41,64-73]. Six signatures were developed in the context of prognostic or diagnostic studies ('prognostic' signatures) while eighteen were reported to monitor the activation status of pathways associated to oncogenesis ('oncogenic' signatures). While being derived independently, each of the signatures can be represented as a set of genes whose up-or down-regulation is associated with a given phenotype, such as the activation/engagement of a pathway or a defined clinical prognosis. Because the clinical phenotype of a tumour is closely related to the underlying biology and ultimately depends on the activation status of a complex network of oncogenic genes and pathways, the distinction between 'oncogenic' and 'prognostic' signatures has to be intended as purely indicative. To provide a metric that can be uniformly applied to all signatures, we defined a simple quantitative 'signature score' by using for each sample the weighted average of the gene expression levels of all genes composing a signature (see Methods). Setting this metric allowed us to investigate all pair wise relationships between different prognostic signatures, both in human and dog tumor samples. For each pair wise comparison the genes common to the respective signature pair were excluded from the computation of Pearson's correlation. This way we insured that signature correlation values were not driven by intrinsically similar gene contents. Starting with the analysis of the prognostic signatures, we observed that most pairs are positively correlated with each other in the human tumor data set (Pearson's correlation > 0.48, P value < 0.0001) (Figures 5 and 7). In some cases, these results reproduce what was also observed elsewhere, regarding the relationships between 'Wound' signature and poor prognosis [41], 'Hypoxia' and poor prognosis [71], Proliferation' and 'Van't Veer' signatures [37].

**Table 1:** Oncogenic and prognostic gene signatures

**Signature**	**Type**	**Model system**
Akt Majumder 2004	Oncogenic	Transgenic mouse prostate over-expressin human AKT1
Androgen Chen 2004	Oncogenic	Treatment of LNCaP prostate cells with R1881
Androgen DePrimo 2002	Oncogenic	Treatment of LNCaP prostate cells with R1881
β-catenin Bild 2006	Oncogenic	Adenovirus infection of human primary mammary epithelial cells (HMECs)
Cell cycle Whitfield 2002	Oncogenic	Synchronized HeLa cell cultures
CyclinD1 Lamb 2003	Oncogenic	Adenovirus infection of MCF-7 breast cancer cells
E2F3 Bild 2006	Oncogenic	Adenovirus infection of human primary mammary epithelial cells (HMECs)
EGFR Creighton 2006	Oncogenic	Stable transfection of MCF-7 breast cancer cells
Hypoxia Chi 2006	Oncogenic	Human cells from different tissues
MAPK Creighton 2006	Oncogenic	Stable transfection of MCF-7 breast cancer cells
MEK Creighton 2006	Oncogenic	Stable transfection of MCF-7 breast cancer cells
Myc Bild 2006	Oncogenic	Adenovirus infection of human primary mammary epithelial cells (HMECs)
Myc Coller 2000	Oncogenic	Conditional Myc-estrogen receptor fusion protein in human primary fibroblast cells
Raf1 Creighton 2006	Oncogenic	Stable transfection of MCF-7 breast cancer cells
Ras Bild 2006	Oncogenic	Adenovirus infection of human primary mammary epithelial cells (HMECs)
Src Bild 2006	Oncogenic	Adenovirus infection of human primary mammary epithelial cells (HMECs)
Tamoxifen Chanrion 2008	Oncogenic	Human primary breast tumors
PTEN Saal 2007	Oncogenic	Human primary breast tumors
70 poor prognosis van't Veer 2002	Prognostic	Human primary breast tumors
ERBB2 Creighton 2006	Prognostic	Stable transfection of MCF-7 breast cancer cells
Grade 1–3 Ivshina 2006	Prognostic	Human primary breast tumors
Proliferation signature Dai 2005	Prognostic	Human primary breast tumors
Wound Chang 2004	Prognostic	Response to serum exposure in fibroblasts from ten anatomic sites

**Figure 8:**
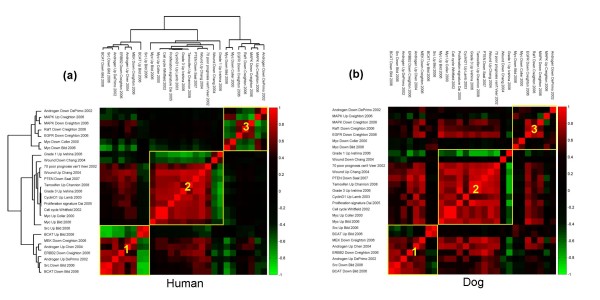
**Correlation between prognostic and oncogenic signatures**. Heat map representation of the pair wise Pearson's correlations between a selection of "oncogenic" and "prognostic" signatures described in Table 1. (a) Hierarchical clustering of signatures based on the pair wise correlation in human tumor samples. (b) Pair wise Pearson's correlations in the dog tumor dataset are represented. Signatures are ordered as in (a). In yellow are highlighted the three major clusters of correlated signatures described in the text.

Similar relationships were observed in the dog tumor samples, again underlining the close resemblance of the dog tumor samples to their human counterparts (Figures 5 and 7). To establish how prognostic and oncogenic signatures are related in the two species, the same analysis was extended to the complete set of signatures of the compendium, including now also the 'oncogenic' signatures. Each signature was split into the up- and the down-regulated arm and, after computing all pair wise correlations independently for human and dog samples, signature were clustered accordingly and the results represented in the form of squared heat maps (Figure 8 and Additional files 7 and 8). Coherent clusters of closely correlated signatures emerged, visible in tumours of both species. Looking more closely at the results, it can be noticed that signatures monitoring the same pathway, for example 'Myc' and 'Response to androgens' represented by two independently developed signatures, cluster close to each other, as expected.

Three major clusters of signatures showing close reciprocal correlations are visible in both tumor types (Figure 8). 'Down', or 'Up' arms of the 'SRC',' B-catenin', 'MEK', 'ERBB2' and 'androgen' (both arms) signatures appear in cluster 1 in agreement with previous studies on the respective pathways. For example, *ERBB2 *and MEK (*MAP2K1 *and *MAP2K2*) are part of the larger 'MAPK pathway' [70] related to the response to androgens [74] and it is therefore unsurprising that these three signatures appear to be co-regulated. Likewise, the 'SRC' and 'B-catenin' pathways are known to be co-regulated in breast cancer and associated with poor survival [67,75]. Most signatures initially defined as 'prognostic' ('Van't Veer', 'Grade1-Grade3', 'Proliferation', 'Wound'), appear in cluster 2, together with additional signatures such as 'Cell Cycle', 'Cyclin D1', 'Resistance to Tamoxifen' and 'PTEN'. This cluster therefore mirrors the reciprocal association between loss of *PTEN *and cancer progression, cell cycle, poor prognosis and resistance to Tamoxifen [37-41]. Moreover, the presence of both the 'Myc' and the 'Wound' signature in cluster 2 is consistent with the notion that activation of the Wound signature in primary breast cancer is prominently associated with over-expression of *MYC *due to gene on chromosome arm 8q [76]. The 'ERBB2' signature, although present in cluster 1, nevertheless shows strong correlation with other prognostic signatures, as expected for the important role of *ERBB2 *over-expression in cancer prognosis [77]. Cluster 3 contains co-regulations of the 'Response to androgens' and 'MAPK' signatures, whose corresponding pathways are also reported to be correlated [74]. In addition, the 'Raf1', 'EGFR' and 'E2F3' signatures also fall into this cluster, confirming their known associations with the MAPK pathway[74]. Most importantly, the same reciprocal pair wise correlations were maintained in the two species, providing again strong evidence for a conserved relationship between the underlying networks of genes related to the establishment, progression and clinical outcome of breast tumors.

## Discussion

We present the first genome-wide comparative analysis of transcriptional changes occurring in spontaneous mammary tumors of human and dog. Accumulation of multiple mutations and alterations in cancer genomes promotes the deregulation of individual genes and complex cell-signaling pathways controlling essential cellular functions such as proliferation, differentiation and apoptosis. To assess the extent to which deregulation of these processes are similar in canine and human mammary tumors, we have used gene expression analysis following an "incremental" approach. Starting from the analysis of individual genes, the analysis was extended to explore global perturbations in gene pathways and ultimately to establish global relationships between large sets of cancer related gene signatures. Shifting the focus from individual genes to pathways and gene signatures allows for a more comprehensive and interpretable view of human and dog tumors. In addition, it compensates for the limited resolution of the dog data set, caused by the reduced sample size and by the intrinsically lower quality of the canine microarray. In fact, more recently assembled genomes like that of dog tend to have a lower quality of gene annotation translating into lower microarray data quality due to missing or incorrectly designed gene probes. By using a high-level multi-gene-focused analytical approach we believe we have overcome many of these potential limitations. Our analysis provides a global picture of oncogenic pathway deregulation and establishes a relationship between a large panel of cancer-related gene signatures in tumors of both species. By applying principal component cluster analysis we have shown the global transcriptional profiles of both tumor types to be dominated by their tumor/normal origin and, only to a minor degree, by their species membership. This analysis also revealed clear evidence for a close similarity between human and dog tumors regarding the perturbation of many cancer-related gene sets and pathways. Examples are 'PI3K/AKT', 'KRAS', 'PTEN', 'WNT-beta catenin' and 'MAPK signaling' pathways, as well as a group of genes specific of human tumor initiating cells (CD44+CD24-). Since pathway deregulation is closely linked to sensitivity to therapeutic agents targeting components of the pathway [67], our data emphasize the high potential value of the dog as a preclinical model to test therapeutic agents targeting pathways commonly deregulated in mammary tumors of the two species. Importantly, the close similarity observed between the tumors should also greatly facilitate the development of biomarkers to evaluate and measure drug response. An ultimate proof will however have to await experimental data confirming that signatures developed in dog maintain their predictive value in a human cancer setting. In the present study we tried to provide a preliminary answer to this question by systematically exploring the expression level of a large set of breast cancer related gene signatures derived from human tumors in samples from dog mammary tumors. The advantage of such an analytical approach is two-fold: firstly, it allows the unbiased discovery of new relationships between different oncogenic and prognostic transcriptional signatures; secondly, it opens the possibility of assessing on a high level how relationships and deregulations of pathways and signatures are maintained across difference cancer settings. Our results show that most of the prognostic signatures are strongly correlated with each other, both in human and dog tumors. Despite different gene compositions and different approaches used for their development, these signatures apparently track common sets of biological features present in tumors of both species. We further show that relationships observed in the human tumors, e.g. activation of an oncogenic pathway and prognostic outcome, can be monitored by the respective gene signatures conserved in the dog tumor samples. This suggests the same networks of signaling circuitries govern the biology of mammary cancer in the two species and that signatures derived from human breast tumors are likely to monitor equivalent biological phenomena in the corresponding canine tumors. Importantly, our data also suggest the possibility of the reverse path, i.e. the development of transcriptional biomarkers in dogs to be applied subsequently to humans.

## Conclusion

These results provide the basis for considering spontaneous canine mammary tumors as a valuable and predictive model for human breast cancer. If confirmed by additional experimental efforts, this data would open up the possibility to perform in a more systematic and, most importantly, more predictive way, the preclinical development of cancer therapeutics and associated biomarkers of drug response.

## Methods

### Canine and human mammary samples

The canine dataset consists of 26 tumors (17 malignant, 5 benign and 4 intermediate) and 7 normal mammary glands from 10 different donors. Tumors derived from the same animal were always extracted from different glands. Detailed pathological information for each canine sample are available in Additional file 1.

The 33 canine mammary tissues samples were classified applying the diagnostic criteria proposed by the World Health Organization (WHO) [19] while histological grading was performed according to [78]. Therefore, the overall grade was obtained assessing three morphological features: degree of tubular formation, degree of nuclear and cellular pleomorphism, and mitotic count. Morphological diagnosis allowed identification of normal mammary glands (7 samples), hyperplastic/dysplastic lesions (3 samples), adenomas (2 samples), and malignant tumours (17 samples). All samples were snap-frozen in liquid nitrogen after collection.

Samples were assigned to 4 different classes, as follows (see Additional file 1): Class 0: 'normal' samples; Class 1: 'hyperplastic/dysplastic lesions' (representing benign proliferative lesions) and 'adenomas' (benign tumors); Class 2: 'in situ carcinomas' (malignant tumor with a better prognosis as compared to other malignant lesions) and 'tubular carcinomas' (showing well differentiated morphology and no evidence of infiltration); Class 3: 'simple carcinomas' and 'complex carcinomas' (representing aggressive forms of malignant tumors). Classes 0, 1, 2 and 3 are respectively referred in the text as 'normal', 'benign', 'intermediate' and 'malignant' samples.

The human dataset included 68 infiltrating ductal mammary carcinoma and 61 adjacent non-involved tissues. After surgical removal, only samples with greater than 70% tumor cells were retained. Information regarding tumor stage and percentage of tumor cells are provided in Additional file 2. All samples were snap-frozen in liquid nitrogen after collection. Human samples were purchased from Genomics Collaborative, Inc., which was acquired by SeraCare Life Sciences. The samples were appropriately consented for the purposes of our study, in compliance with the Helsinki Declaration  (see Additional files 9 and 10).

Collection of dog samples was submitted for approval to IRBM IACUC (Institutional Animal Care & Use Committee) and then to the Italian Regulatory Agency for approval (Italian Ministry of Health). IRBM is in compliance with European Legislation for the Protection of Animal Used for Experimental & Scientific Purposes (EEC 86/609) and received FULL AAALAC Accreditation with no. 1242. All samples were obtained from client-owned bitches undergoing routine mastectomy. To obtain the histological samples, no experimental protocols were applied. Nevertheless, a written informed consent was obtained from all dog owners.

### Microarray experiments

RNA was isolated, labelled and hybridized as in [40]. Briefly, total RNA was isolated with RNAzolB and finally dissolved in RNase-free water. Then 25 μg of total RNA was treated with DNase using the Qiagen RNase-free DNase kit and RNeasy spin columns. Total RNA was dissolved in RNase-free water to a final concentration of 0.2 μg/μl. cRNA was generated by in vitro transcription using T7 RNA polymerase on 5 μg of total RNA and labelled with Cy5 or Cy3 (Cy Dye, Amersham Pharmacia Biotech). 5 μg of labelled-RNA from each sample were co-hybridized with 5 μg of a normal reference pool, consisting of an equal amount of cRNA extracted from mammary healthy samples of the corresponding species.

Labelled cRNAs were fragmented to an average size of 50–100 nucleotides by heating the samples to 60°C with 10 mM of zinc chloride and then adding an hybridization buffer containing 1 M NaCl, 0.5% sodium sarcosine, 50 mM MES, pH6.5, and formamide to a final concentration of 30%. The final volume was 3 ml at 40°C.

The human samples were hybridized on a Human 25K array containing 23,720 unique probes for ~21,000 human genes. Canine samples were hybridized on a Dog 44k array containing 39,558 unique probes corresponding to ~30,000 canine genes. Based on 3' end distance and cross-hybridization in-silico assessment, potentially unreliable probes were removed from the analysis. This filter yielded a final number of 28,541 and 17,888 probes for dog and human respectively. The selection of reliable probes was done using the genomic assembly hg18 and canFam2 for human and dog respectively. The array designs Human 25k (v 3.0 A1) and Dog 44k 1.0 were submitted to GEO with accessions GPL3991 and GPL7198 respectively. Both human and dog arrays were manufactured by Agilent (Agilent Technologies Inc., Santa Clara, CA, USA). The probes were 60 bp in length and were selected based on the oligonucleotide probe design program [79]. Each sample was hybridized in duplicate with fluor reversal to systematically correct for dye bias. After hybridization, slides were washed and scanned using a confocal laser scanner (Agilent Technologies). The intensities obtained after scanning were quantified, background-corrected and normalized. Expression changes between each sample (tumours and normals) and the corresponding normal reference pool were quantified as the log_10 _of the expression ratio. The Rosetta error model [80] was applied to dye-swapped pairs of hybridizations to compute a weighted average ratio per gene and the corresponding p-value indicating the probability a gene is falsely classified as deregulated. All microarray data were submitted as a unique data series to GEO, with accession GSE14999.

### Clustering of canine mammary samples

Prior to clustering the dog mammary dataset was filtered for non variable genes by removing those genes that showed significant changes in expression (fold-change > 3 or < 1/3) in less than 3 samples. For each gene, fold change was calculated as the ratio between each sample and the corresponding normal reference pool. Therefore for human samples the fold change was the ratio against the pool of human normal samples while for the dog samples the fold change was the ratio against the pool of dog normal samples.

Using these criteria, 2,008 genes were selected for an average linkage hierarchical clustering based on Pearson correlation coefficients. The overrepresentation of normal, benign-intermediate and malignant samples in each cluster was assessed by hypergeometric test.

### Identification of group-specific genes in canine mammary samples

Starting from the whole set of 28541 reliable dog probes, we selected the genes differentially expressed among the three groups of samples (M, malignant; I-B, intermediate and benign; N, normal) by applying one-way ANOVA test on gene expression ratio values, calculated against the reference pool of normal samples. The resulting p-values were corrected for multiple testing by converting them into the corresponding q-values using the *qvalue *package [81] of the R statistical programming language [82]. The q-value corresponds to the false discovery rate (FDR). Therefore a q-value threshold of 0.001 implies that 0.1% of the genes identified as differentially expressed are false positive. Using the conservative q-value cutoff of 0.001 we selected 1043 genes (Additional file 3). The selected genes have then been submitted to t-test, in which genes were individually tested for up- or down-regulation in each class of samples against the others. In this way it was determined in which sample class each selected gene was found to be differentially expressed. Genes could therefore be classified in 6 groups, according to their pattern of expression. For display purposes, the groups of samples (M, I-B and N) and genes (A to F) were clustered separately using an average linkage hierarchical clustering and the Pearson correlation as similarity measure.

### Combining human and dog datasets

To compare human and dog datasets, we have identified the set of genes orthologous between the two species. Based on conservative criteria (E-value of homolog relationship < 1E-10; the homologous gene is the best BLAST match in both species; conserved synteny), we defined a set of 9,963 orthologs (the ratio Tumor vs Pool of Normal, the error and the corresponding p-value for the combined dataset are available as Additional files 11, 12 and 13). Prior to combining the dog and human datasets for hierarchical clustering, we selected the subset of most variable genes having a three fold-change (or greater) in at least 3 human and dog samples. This filter yielded 600 orthologs. Genes were then standardized (mean 0 and standard deviation 1) independently in each dataset. Finally, the combined dataset was clustered using an average linkage algorithm based on Pearson correlation coefficients.

### Identification of tumour deregulated genes

Differences in average gene expression between the set of malignant tumours and normal samples were computed independently in the human and canine datasets by t-test. P-values were adjusted for multiple comparisons and genes differentially up- or down- regulated compared to the normal counterpart (q-value < 0.001) were identified. Canine samples classified as intermediate or benign were removed from the analysis.

### Gene set enrichment analysis

Groups of genes identified in previous steps were compared to annotated gene sets in order to identify the functional classes that were significantly over-represented. Enrichment p-values were computed according to the Fisher's exact test. A total number of about 12,000 annotated gene sets were obtained from publicly available sources (Gene Ontology [83], KEGG [84], Interpro [85], Panther [86], oPOSSUM [87]), gene sets of relevance to cancer taken from published references [37,39,44,45,68,88-92]) and commercial sources (GeneGo (GeneGo Inc., St Joseph, MI, USA), Ingenuity (Ingenuity Systems Inc, Mountain View, CA, USA), TRANSFAC [93]).

In light of a number of considerations, we decided to use and report the uncorrected p-values and do not correct for multiple testing. First, the effective number of gene sets effectively taken into account to generate the presented results is significantly lower the complete set used to run the automated analysis. Second, there is a very high degree of overlap between different gene sets. Third, as we also demonstrated in the signature correlation analysis, many gene sets are closely related to each other in terms of transcription. As a consequence, single tests performed on the individual gene sets are strongly dependent on each other, violating the assumption of independence required by standard correction methods such as 'Bonferroni', 'Holm' and 'FDR'. Thus in this context, standard correction for multiple testing would have resulted as too conservative. To be noticed also that most of the discussed pathways have a p-value much lower than the standard threshold of 0.05.

### Computation of the pair wise correlations between prognostic and oncogenic signatures

We analyzed the expression patterns of 24 published transcriptional signatures of breast cancer collected from different studies (see Table 1 for details). All signatures include genes up- and down-regulated. A signature is then composed of one or two 'arms', containing perturbed genes either down- and up-regulated. In the analyses shown in Figures 5 and 7 a global 'signature score' was assigned to each sample by using the weighted average of the gene expression level of all the genes of the signature. For each gene, the weight was respectively -1 and +1 depending whether the gene was part of the down- or the up-regulated arm of the signature. In the analysis of Figure 8, when signatures were composed of two arms, individual arm scores were computed and analyzed separately for each sample, by averaging the corresponding gene expression levels.

In all correlation analyses pair wise Pearson correlations between signatures were computed independently for the human and dog datasets, after removing the normal samples. For each signature pair, genes in common were excluded from the computation of the scores. In this way we insured that observed correlations could not be ascribed to a similar gene composition of the different signatures.

In Figure 8 all signature arms were clustered using the calculated correlation values and the clustering results were represented as squared heat maps.

## Authors' contributions

PU performed data analysis and participated to drafting the manuscript. LA, JW and ALO participated to experimental design and data interpretation. AK and JC designed the dog array layout. FP and VV carried out the preparation of canine RNA samples. GM and LM coordinated activities for the collection of canine mammary tumor samples. VZ and FA performed the histopatological annotation of the dog samples. GC and AL participated in the conception and the design of the study. NLM participated in the conception and the coordination of the study. EDR conceived the study, coordinated data analysis and wrote the final version of the manuscriptAll authors read and approved the final manuscript.

## Supplementary Material

Additional file 1 **Description of 33 canine mammary samples**. Pathological information and classification (normal, benign, intermediate, malignant) of 33 canine mammary samples extracted from 10 different animals. Malignant mammary tumours were further classified based on the tumour aggressiveness in M1 (infiltrating/solid), M2 (simple) and M3 (complex). Click here for file

Additional file 2 **Description of 129 human mammary samples**. Information regarding sample classification, tumour size, tumour stage and percentage of tumor cells of the 129 human mammary samples. Click here for file

Additional file 3 **List of 1043 group-specific genes selected by ANOVA in dog dataset**. – worksheet '1043 AN0VA': genes identified as differentially expressed among the three groups of dog mammary samples (M, malignant; I-B, intermediate-benign; N, normal) (ANOVA, q-value < 0.001). – worksheet 'enrichment analysis': results of the gene set enrichment analysis separately run on each of the six groups identified based on their pattern of expression. Click here for file

Additional file 4 **Identification of genes most correlated to the 1st principal component and results of gene set enrichment analysis**. Results of the gene set enrichment analysis of the genes most correlated to the 1^st ^principal component. The correlations of the genes with the 1^st ^principal component were transformed to SDs from the mean, then genes with values > 1.5 (positive correlation) or < -1.5 (negative correlation) were selected. These genes were analyzed separately by gene set enrichment analysis. Click here for file

Additional file 5 **Tumour deregulated genes in human and dog datasets**. Genes identified as differentially expressed between tumor and pool of normal samples in human and dog datasets (t-test, q-value < 0.001). Click here for file

Additional file 6 **Gene set enrichment analysis of tumor deregulated genes**. Results of gene set enrichment analysis of tumor deregulated genes in human and dog. Gene sets that were enriched (p < 0.05) in at least one dataset are shown. Click here for file

Additional file 7 **Correlation between prognostic and oncogenic signatures in human breast tumors**. Graphical representation of the pair wise Pearson correlation in the human tumor dataset between all the "oncogenic" and "prognostic" signatures described in Table 1. Signatures are ordered by agglomerative hierarchical clustering based on Pearson coefficients. Click here for file

Additional file 8 **Correlation between prognostic and oncogenic signatures in canine mammary tumors**. Graphical representation of the pair wise Pearson correlation in the canine tumor dataset between all the "oncogenic" and "prognostic" signatures described in Table 1. Samples are ordered according to the hierarchical clustering computed on human tumors (Additional file 9). Click here for file

Additional file 9 **Consent guidelines**. Guidelines followed by Genomics Collaborative, Inc. for obtaining patients informed consent. Click here for file

Additional file 10 **Consent Module**. Informed consent module delivered by Genomics Collaborative, Inc to patients who contributed to the collection human tissue samples. Click here for file

Additional file 11 **Log10 of the ratio Tumor vs Pool of Normal for 9,963 dog-human orthologous genes**. Contains a tab-delimited file with the normalized log_10_ratio for the 9,963 dog-human orthologous genes across the 162 samples. Click here for file

Additional file 12 **Error associated to the log10 of the ratio Tumor vs Pool of Normal for 9,963 dog-human orthologous genes**. Tab-delimited file with the error associated to the log_10_ratio for the 9,963 dog-human orthologous genes across the 162 samples. Click here for file

Additional file 13 **Rosetta p-value associated to the log10 of the ratio Tumor vs Pool of Normal for 9,963 dog-human orthologous genes**. Tab-delimited file with the Rosetta p-values of differential expression for the 9,963 dog-human orthologous genes across the 162 samples. Click here for file
