# Supplementary figures and images for: Comparative expression pathway analysis of human and canine mammary tumors
Source: BMC Genomics. 2009 Mar 27;10:135. doi: 10.1186/1471-2164-10-135 (PMC2670324; doi:10.1186/1471-2164-10-135)

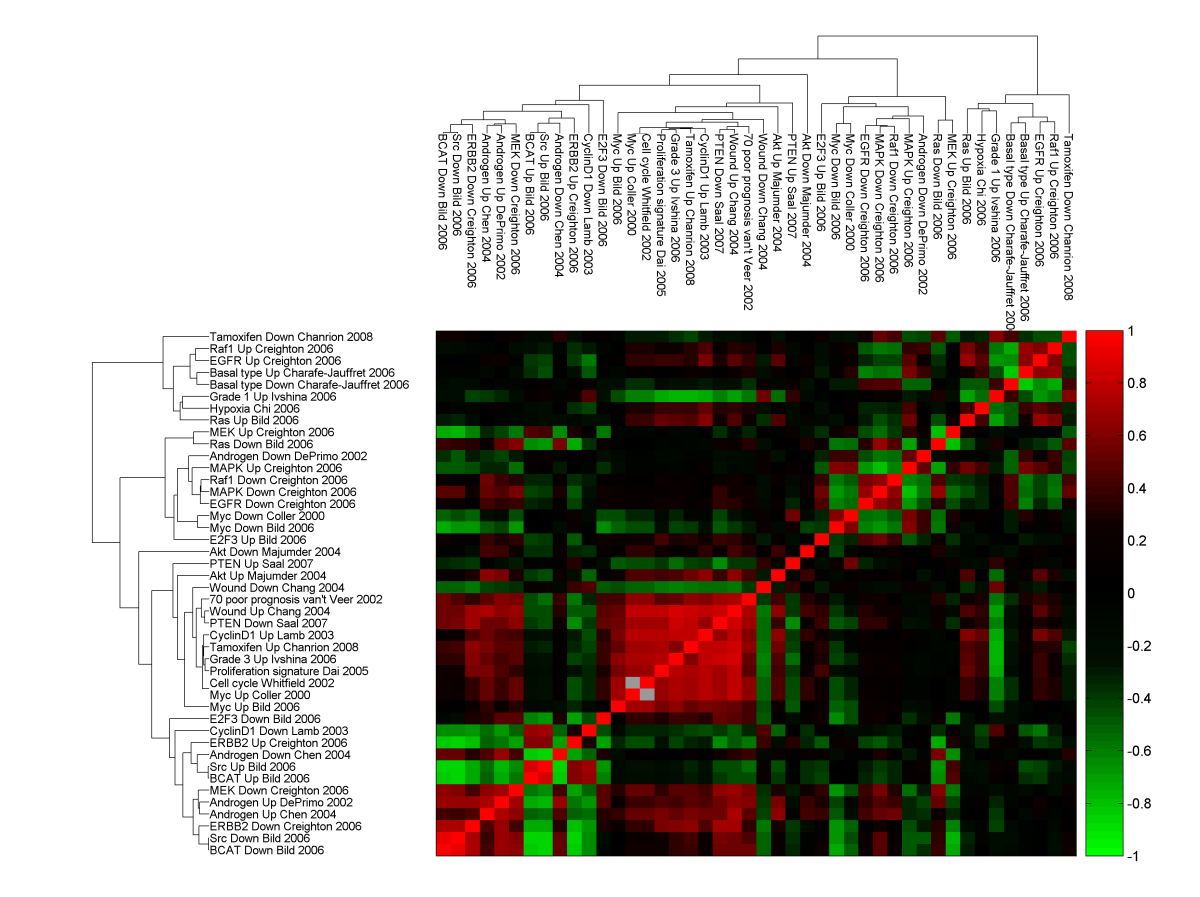

Supplement: Additional file 7 — Correlation between prognostic and oncogenic signatures in human breast tumors. Graphical representation of the pair wise Pearson correlation in the human tumor dataset between all the "oncogenic" and "prognostic" signatures described in Table 1. Signatures are ordered by agglomerative hierarchical clustering based on Pearson coefficients. [file 1471-2164-10-135-S7.tiff]

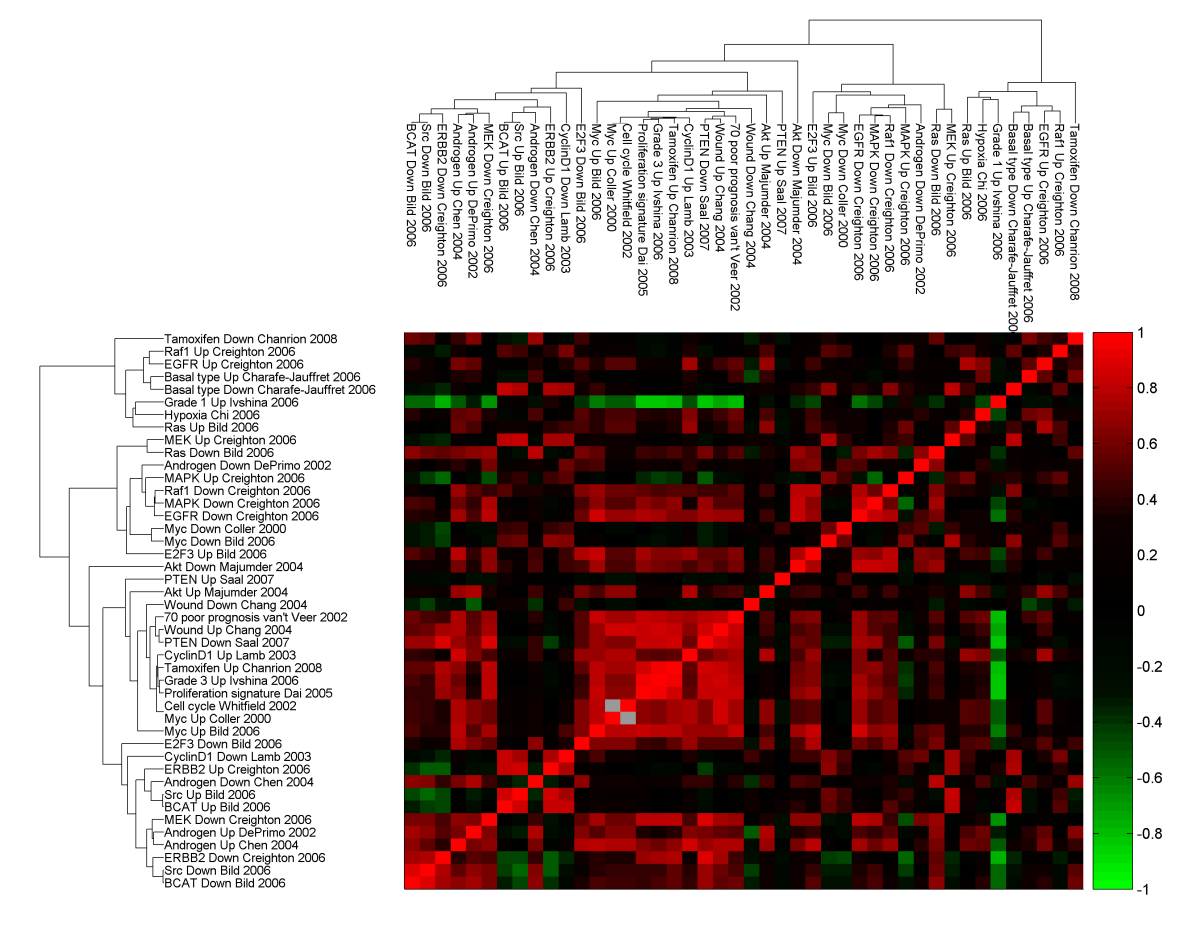

Supplement: Additional file 8 — Correlation between prognostic and oncogenic signatures in canine mammary tumors. Graphical representation of the pair wise Pearson correlation in the canine tumor dataset between all the "oncogenic" and "prognostic" signatures described in Table 1. Samples are ordered according to the hierarchical clustering computed on human tumors (Additional file 9). [file 1471-2164-10-135-S8.tiff]
