# Supplementary material for: Comparative expression pathway analysis of human and canine mammary tumors
Source: BMC Genomics. 2009 Mar 27;10:135. doi: 10.1186/1471-2164-10-135 (PMC2670324; doi:10.1186/1471-2164-10-135)
Supplement: Additional file 9 — Consent guidelines. Guidelines followed by Genomics Collaborative, Inc. for obtaining patients informed consent. [file 1471-2164-10-135-S9.doc]

# Guidelines for Obtaining Informed Consent

The consent process is one of the most important parts of planning a research proposal since the central requirement for human subjects research is that people participate voluntarily. The process ensures that the potential subject understands the study, its risks and benefits and verifies his or her willingness to participate.

The **PROCESS** of obtaining informed consent is different from the consent form in that: It involves **meeting** with a potential subject and finding out whether he or she is capable of giving consent. It involves **discussing** the purpose, risks, and benefits of participation. **Administering** the consent form then formalizes the agreement with the participate and is designed to document the process.

If consent is to be informed, the subject must genuinely understand the study. At the end of the process, subject should be able to **verbalize** what they are consenting to.

Guidelines for explaining the Genomics Collaborative study

All information that is required by federal regulations is included in the Genomics Collaborative consent form. However you should be sure that the subject understands the following points:

Research purpose and use of samples:

- The purpose of the study is to build a large bank of tissue and DNA samples, contributed by hundreds of thousands of people, for use by researchers around the world in the study of genetic diseases.
- Their sample will be used by many researchers in many projects over many years, but Genomics Collaborative will not allow researchers to obtain samples for unethical research, such as human cloning or fetal research.
- Participation is voluntary. Refusal to participate involves no penalty or decrease in benefits to which the subject is otherwise entitled.

Confidentiality and Privacy:

- Since Genomics Collaborative uses a system of barcodes to identify samples, Genomics Collaborative never sees the name of the person who contributes the samples. No one using these samples will EVER know the subject’s identity.
- As part of the study, the subject’s medical record will be accessed, but only by qualified healthcare personnel (if it is yourself, you should tell them this). Nothing will be placed in their medical record concerning this study (unless your IRB requires otherwise).

Benefits and Compensation:

- The research for which these samples will be used may (MAY, not WILL) result in the discovery of an important test or a treatment. If a company or researcher develops a produce from this research and makes money, the subject will not be entitled to receive any money.

Contact Information:

- Clearly identify to the subject the names of people listed on the consent form who can be contacted to answer questions about the research. This will include the Principal Investigator, the IRB that can explain the rights of research subjects, and the contact person in the event the subject suffers injury related to the research.

Assessing the subject’s understanding

The burden of ensuring that someone who might participate genuinely understands the research falls upon you, not upon the prospective subject. Hence it is critical to the consent process that you not only field questions, but also ask questions.

Useful questions to assess the subject’s understanding should be open-ended and non-directive.

Examples of open-ended questions are:

- "Just so that I'm sure you understand what is expected of you here, would you please explain to me what you think we're going to ask you to do?"
- "Describe in your own words the purpose of the study."
- "What more would you like to know?"
- "What is the possible benefit to you for contributing a tissue and blood sample to this study?
- “What are the possible risks?"

Avoid closed-ended questions such as:

- "Do you understand?"
- "Do you have any questions?"
- "Do you see that there are some risks to participating in this study?

Documenting the subject’s consent with a signed consent form

Once a subject understands the study and has expressed a willingness to participate, you must document the subject’s consent. Have the subject sign and date the consent form. .

Once the form is signed by the subject and by you in a secure location (not in the subject’s medical record unless required by your IRB).

THE BARCODE LABELS USED TO IDENTIFY THE CASE REPORT FORM AND THE TISSUE AND BLOOD SAMPLES MUST NOT BE PLACED ON THE CONSENT FORM. Doing so would create a link between the subject’s name and the barcode and could cause their sample to be identified.
