# Supplementary material for: Comparative expression pathway analysis of human and canine mammary tumors
Source: BMC Genomics. 2009 Mar 27;10:135. doi: 10.1186/1471-2164-10-135 (PMC2670324; doi:10.1186/1471-2164-10-135)
Supplement: Additional file 10 — Consent Module. Informed consent module delivered by Genomics Collaborative, Inc to patients who contributed to the collection human tissue samples. [file 1471-2164-10-135-S10.doc]

# Consent to Participate in a Clinical Study and Contribute a Blood and Tissue Sample

# Title: Anonymized Collection of Participant Tissue, Blood and Phenotypic Data for Inclusion in a Repository and use in Genomic (from DNA and RNA), Serologic and Metabolic (from Serum) and Proteomic (from Protein) Research Studies

# Study Doctor:

**Address:**

**Phone No.:**

**What is the purpose of this study and what is Genomics Collaborative?**

You have been asked to participate in this research study because you are scheduled to have or have just had surgery to remove tissue from your body. The purpose of this study is to collect tissue and blood samples to be used in research to find genes, or the products of genes such as RNA or protein, that may be involved in diseases like cancer, heart disease and diabetes. This study is a one-time collection.

Genomics Collaborative is a division of SeraCare Life Sciences, which is a publicly held company. Genomics Collaborative has a large repository (collection) of human biological materials such as DNA, RNA and Protein, which were obtained from many people around the world who also participated in this study and contributed tissue and blood samples. DNA is the molecule present in plant and animal cells that carries genetic (inherited) information. This information (like eye color or whether you are likely to develop a disease) is passed from one generation to the next. The information coded in DNA is translated in to eye color or a disease through a messenger molecule, called RNA, which specifies how many and what types of protein a cell should make. Your tissue and your blood cells contain DNA as well as RNA, protein and metabolites. You are being asked to contribute samples of your tissue and blood so that Genomics Collaborative can store and provide samples that may include DNA, RNA and tissue, serum or plasma for studies of proteins and metabolites.

Genomics Collaborative collects tissue and blood samples from many hospitals. Your sample and many others then become part of Genomics Collaborative’s repository. This repository will contain tens of thousands of samples that can be categorized by common human diseases. The repository’s samples are stored in a way that is useful for long-term, repeated use. Genomics Collaborative then makes its repository available to researchers from a variety of organizations, such as pharmaceutical companies and universities (as well as scientists within Genomics Collaborative), for use in their studies searching for genes and proteins involved in these diseases. These scientists will pay a fee to use these samples and the medical information about the samples.

# What is going to happen and how long is it going to take?

If some of the tissue removed during your surgery is not needed to diagnose your condition, it will be frozen and transferred to Genomics Collaborative’s repository. In addition you will be asked to contribute a blood sample. For the blood draw, a needle will be used to collect about 3 or 4 tablespoonfuls of blood from a vein in your arm. An anonymous questionnaire about your background and medical history will be completed. After the questionnaire is done, the person who worked with you on the questionnaire will review your medical record. He or she will confirm information you have given. Only medical information will be reviewed. Nothing you say will be entered into your medical record. The interview and blood draw should take around 20 to 30 minutes.

**What privacy will I have?**

This study will be conducted in a confidential manner. Only authorized study personnel assigned by your study doctor will handle your samples and questionnaire before they are packaged and mailed to Genomics Collaborative labeled with a unique barcode number, like those used in the supermarket. This unique barcode is not associated with any of you personal information. Genomics Collaborative may send representatives to inspect the study records at [INSTITUTION] and ensure that they are conducting the study in accordance with the written protocol, and all federal and state regulations. The representatives will not be able to link your medical record with the questionnaire or samples sent to Genomics Collaborative. The tissue and blood samples, as well as the questionnaire, sent to Genomics Collaborative will not identify you by name. The only identification on the samples and documents sent to Genomics Collaborative will be this unique barcode.

(INSERT INSTITUTION) will keep all research records that identify you private to the extent allowed by law. However, representatives from the Food and Drug Administration, [INSTITUTION], (INSERT IRB) and Genomics Collaborative may inspect the study records that identify you to ensure accuracy of the data and compliance with the study.

Once your tissue and blood samples and questionnaire are sent to Genomics Collaborative, the tissue and blood samples will be processed, preserved, and stored. The information gathered from the questionnaire and from your medical record will be entered into a computer database. Both your samples and the questionnaire data will be identified by a unique barcode. Genomics Collaborative will not enter any personally identifying information about you into the database. You will be identified only by the barcode assigned to you.

From your one blood sample, Genomics Collaborative will use some of the blood to create DNA samples that may be used by many different researchers, and the remainder of the blood will be stored as frozen serum and RNA samples. Genomics Collaborative may also “grow” some of your blood cells in the laboratory (the cells would grow in number, not size). Genomics Collaborative will then make samples of your DNA, serum, or RNA available to scientists for years after you originally contribute your blood.

From your tissue sample, Genomics Collaborative or other researchers may remove some RNA. Genomics Collaborative will then make samples of your tissue or RNA available to scientists for years after you originally contribute your tissue.

**Will Genomics Collaborative know who I am?**

No. Genomics Collaborative and the scientists who use samples of your blood or tissue will not know your name because the samples can only be identified by the unique barcode. Additionally, scientists will not know which study doctor originally collected your samples. But they may know your age, sex, race, medical history, etc.

Publications resulting from research using your sample will not identify you. It is not possible for Genomics Collaborative to give information about you or your blood or tissue sample to insurance companies, your employer, or other non-scientific organizations since Genomics Collaborative will not know your identity.

**Will I be contacted again?**

### No. Since Genomics Collaborative will not know your name, there will be no way to contact you again.

# How do scientists get my sample?

Before a scientist is allowed to use Genomics Collaborative’s repository, the scientist must attest to following all applicable laws and regulations regarding the use of human biological materials. Scientists are also asked to provide a short summary of the type of research they will be doing. Scientists conducting research pay a fee to use Genomics Collalborative’s repository. They would ask for a certain number of samples from a particular group of people (for example, 50 men who are over age 65 and have a history of high blood pressure. Samples that meet those requirements will be sent to the scientist.) A scientist will not be able to ask for a specific person.

Will Genomics Collaborative send me the results of any research that uses my sample?

Genomics Collaborative does not collect research results from the scientists who use samples from the repository. Additionally, there is no way Genomics Collaborative can contact you. However, Genomics Collaborative will provide updated information about advancements in genetic research to all the study doctors participating in this study.

**What are the risks of participating in this study?**

# Participating in the study adds no additional risks to your operative procedure. The extra tissue removed from your body would be thrown away if Genomics Collaborative did not store it in the repository, so you will be at no physical risk if Genomics Collaborative stores the tissue. There are minor risks and discomforts associated with blood sampling. This includes pain and possibly bleeding and a bruise at the needle site. Occasionally a person feels faint or light headed when their blood is drawn. Rarely an infection develops. If this happens, it can be treated.

**Will I benefit by participating in this study?**

You will not benefit personally by participating and contributing a tissue and blood sample. You will not receive any money from Genomics Collaborative for your samples. However, there may be a general benefit to society. It is possible that the cause of some genetic diseases could be discovered because of research performed through Genomics Collaborative. It is also possible that academic or industrial institutions, including Genomics Collaborative, may make money from commercial application of research results.

# Will this cost me anything?

# There is no cost to you to take part in this study.

**What if I don’t want to do this?**

You are free to provide your tissue and blood samples to the collection or not. If you choose not to participate, your medical care and treatment at the hospital(s) or doctor’s office will not be affected and there will be no penalty or loss of benefits to which you are otherwise entitled.

**If I change my mind, can I have my sample removed from the study?**

This may not be possible since the study doctor only keeps a link between your name and barcode for a short time. Once that temporary link is broken, no one will be able to identify which sample is yours. If you change your mind, contact your study doctor immediately. If the link is still in place, your study doctor will notify Genomics Collaborative and your samples will be destroyed. Once this link is broken, you will no longer be able to have your samples removed from the study. If you think you may change your mind in the future, do not agree to participate in this study.

**Will my samples be used to study any other disease states besides my primary disease state?**

Yes. An important part of this research is to allow for associations, or connections, to be made between diseases and other patient specific information, which may not be apparent at the time of your initial enrollment in the study. Your samples may thus be used for broad-based genetic research for a variety of disease states. The research will not be limited to the disease that resulted in the removal of your tissue and enrollment in this study.

# Who is in charge of this research?

The person responsible for the conduct of this study at (INSERT HOSPITAL NAME) is (INSERT MD NAME AND TITLE) and he may be reached at: (INSERT PHONE). (INSERT NAME) should be contacted if you have any questions, concerns or injury related to this study.

**What are my rights?**

If you have questions relating to human research at (INSERT HOSPITAL), you are free to contact (INSERT NAME & PHONE), Chairman of the (INSERT HOSPITAL) Institutional Review Board. The Institutional Review Board is a committee that reviews research studies to ensure that the rights and welfare of the participants are protected and that the research is carried out in an ethical manner.

# What if I have questions or am injured?

You may call your study doctor if you have any questions about the study, the blood and tissue collection, or if you have a research-related injury. Your study doctor will provide immediate medical care as needed. Your study doctor’s contact information is listed on page one of this consent form.

# CONSENT

# The purpose of the study, the blood and tissue collection, the procedures, the possible risks and discomforts, and the potential benefits that I may experience from contributing a blood and tissue sample have been explained to me. My participation in this study is voluntary, and I am contributing samples of my blood and tissue to the collection. All my questions have been answered. I have read this consent form or it has been read to me. My signature below indicates my consent and willingness to contribute my sample to the collection. I will be given a copy of this signed consent form. By signing this form, I have not given up any of my legal rights.

## **Subject Name (Printed):________________________________________**

**Subject Signature: ______________________________________Time_____ Date_______**

**Study Doctor (or designee)**

**Signature:______________________________________________Time_____ Date_______**
